# Monitoring Plasma Concentrations of Intravenously Administered Fosfomycin to Prevent Drug-Related Adverse Events: A Retrospective Observational Study

**DOI:** 10.3390/antibiotics14060548

**Published:** 2025-05-27

**Authors:** Kathrin Marx, Nina Malmström, Marie Quast, Annette Glas, Ralph Wendt, Martina Kinzig, Fritz Sörgel, Maike Fedders, Thilo Bertsche, Christoph Lübbert

**Affiliations:** 1 Hospital Pharmacy, Hospital St. Georg, 04129 Leipzig, Germany; kathrin.marx@sanktgeorg.de (K.M.); jennifermariequast@web.de (M.Q.); maike.fedders@skc.de (M.F.); 2 Department of Infectious Diseases and Tropical Medicine, Hospital St. Georg, 04129 Leipzig, Germany; nina.malmstroem@sanktgeorg.de; 3 Department of Laboratory Medicine, Hospital St. Georg, 04129 Leipzig, Germany; annette.glas@sanktgeorg.de (A.G.); 4 Department of Nephrology, Hospital St. Georg, 04129 Leipzig, Germany; ralph.wendt@sanktgeorg.de; 5 Institute for Biomedical and Pharmaceutical Research, 90562 Nuremberg, Germany; martina.kinzig@ibmp.net (M.K.); fritz.soergel@ibmp.net (F.S.); 6 Institute of Pharmacology, West German Heart and Vascular Center, University of Duisburg-Essen, 45141 Essen, Germany; 7 Department of Clinical Pharmacy, Institute of Pharmacy, Leipzig University, 04109 Leipzig, Germany; thilo.bertsche@medizin.uni-leipzig.de (T.B.); 8 Drug Safety Center, Leipzig University Medical Center, 04103 Leipzig, Germany; 9 Division of Infectious Diseases and Tropical Medicine, Department of Medicine I, Leipzig University Medical Center, 04103 Leipzig, Germany; 10 Interdisciplinary Center for Infectious Diseases, Leipzig University Medical Center, 04103 Leipzig, Germany

**Keywords:** fosfomycin, therapeutic drug monitoring (TDM), acute kidney injury (AKI), renal failure, side effects, hypernatremia, hypokalemia, *Staphylococcus aureus*

## Abstract

**Background:** Fosfomycin is used as a combination partner for the treatment of severe non-urinary tract infections. Individualized dosing of fosfomycin based on therapeutic drug monitoring (TDM) has the potential to reduce drug-related adverse events (AEs). **Methods:** This retrospective study used routine data from patients receiving intravenous fosfomycin therapy. Plasma concentrations of fosfomycin were categorized into three different ranges: <64 mg/L, 64–128 mg/L, and >128 mg/L. Subsequently, the influence of acute kidney injury (AKI) on reaching the specific plasma concentration ranges and the occurrence of AEs was analyzed. **Results:** The study included 143 patients (median age 73 years, 66.4% male) with fosfomycin plasma measurements. Beta-lactam antibiotics were most frequently used in combination (62.2%), followed by tetracyclines (12.2%), cotrimoxazole (8.1%), and other agents (17.5%). Fosfomycin concentrations were >128 mg/L in 45% (36/80) of patients with normal renal function, 70.4% (38/54) of patients with AKI stages I to III, and 77.8% (7/9) of patients with renal replacement therapy. AEs occurred in 54% (77/143), mainly hypernatremia (42.6%), hypokalemia (39.9%), and gastrointestinal symptoms (19.6%), with the median fosfomycin plasma concentration being significantly higher in patients with AEs (158 mg/L vs. 131 mg/L, *p* = 0.01). Multivariate logistic regression analysis revealed that patients aged ≥70 years (OR 3.70, 95% CI 1.24–11.5; *p* = 0.02) and patients with fosfomycin plasma concentrations > 128 mg/L (OR 3.30, 95% CI 1.09–10.4; *p* = 0.04) had a higher risk of AEs. **Conclusions:** There was a significant association between high plasma exposure and the occurrence of AEs. In particular, the impact of acute renal insufficiency on fosfomycin plasma concentrations should be considered. Individualized fosfomycin dosing based on TDM and the intensive monitoring of renal function contribute to reducing drug-related side effects.

## 1. Introduction

Fosfomycin was first isolated from streptomycetes (*Streptomyces fradiae*, *Streptomyces viridochromogenes*, and *Streptomyces wedomorensis*) in 1969 [1]. It has a bactericidal effect on proliferating cells and is the only known epoxide antibiotic. Owing to its low molar mass of 138.06 g/Mol and its good water solubility with low plasma protein binding, fosfomycin has good tissue compatibility. It reaches clinically relevant concentrations in serum, soft tissue, lungs, bones, abscesses, cerebrospinal fluid, and heart valves [2,3,4]. The effect of fosfomycin is based on the inhibition of an enzyme-catalyzed reaction in the first step of cell wall synthesis and the associated loss of the cell wall integrity of the bacteria [5]. Some in vitro studies have shown a high mutation rate and associated rapid development of resistance [6,7,8,9]. Combination therapy with another antibiotic, especially beta-lactams, is recommended to counteract the rapid development of resistance to fosfomycin and to take advantage of its synergistic effects with other antibiotics [10,11].

Fosfomycin is available for oral administration as fosfomycin trometamol and for intravenous administration as fosfomycin disodium salt. The intravenous formulation was approved in 2004 [10]. The use of fosfomycin may be limited by adverse events (AEs), in particular, electrolyte disturbances, gastrointestinal intolerance, or heart failure due to sodium overload when administered intravenously [5,10,12,13]. In addition, there is wide variability in dosage, ranging from 12 to 24 g per day, depending on the source and severity of the infection, with the possibility of dose adjustment according to renal function [3,10,14,15].

Individualization of fosfomycin dosing based on therapeutic drug monitoring (TDM) of plasma concentrations helps to minimize the potential development of antibiotic resistance and reduce drug-related AEs [16]. The challenge here is to define the pharmacokinetic/pharmacodynamic (PK/PD) parameters of fosfomycin. There is currently no international consensus on whether bacterial killing by fosfomycin is time- or concentration-dependent [3,4,5,17,18,19,20,21,22]. In a study by Lepak et al. using a validated murine model and a dose fractionation study design, a mean 24 h area under the plasma concentration–time curve (AUC)/minimum inhibitory concentration (MIC) ratio (AUC/MIC_24_) target of 23 and a 1-log kill at 83 were observed in the Enterobacterales group [19]. A Monte Carlo simulation for dose optimization in the treatment of complex osteoarticular infections was based on a PK/PD target of 70% of the time above the minimum inhibitory concentration (T > MIC) [17].

Due to its broad-spectrum activity and excellent tissue penetration, fosfomycin is considered in our hospital as a first-line treatment in specific clinical situations. The main indications in which it plays an important role are the treatment of infective endocarditis, bone and joint infections, complicated skin and soft tissue infections, and bacteremia. To improve bacterial killing and prevent the development of resistance, intravenous fosfomycin is administered only as part of combination therapy. To optimize therapeutic outcomes and avoid side effects, we introduced routine TDM measurements. We decided to measure trough levels because they are easy and reliable to determine and interpret in clinical practice.

The aim of this study was to determine the plasma concentrations of intravenously administered fosfomycin reached with the standard dose and to evaluate these using different concentration ranges. In particular, the effects of acute kidney injury (AKI) on fosfomycin plasma concentrations and the occurrence of AEs were analyzed. In addition, clinical and microbiological responses were evaluated.

## 2. Results

### 2.1. Demographic and Clinical Characteristics of the Patients

Baseline characteristics of the patients are summarized in Table 1 and Table A1. Fosfomycin plasma concentrations were measured in 143 patients (median age 73 years, 66.4% male). Three groups were formed to investigate the causes and effects of the different fosfomycin plasma concentrations: <64 mg/L, 64–128 mg/L, and >128 mg/L. All patients received fosfomycin in addition to at least one other antimicrobial agent, which was selected based on the type of infection and individual patient circumstances. Beta-lactam antibiotics were used most frequently (62.2%), followed by tetracyclines (12.2%), cotrimoxazole (8.1%), and other agents (17.5%). The most common sites of infection were bones and joints (64/143, 45.1%) and skin and soft tissues (24/143, 16.9%). The clinical success rate was assessed by the absence of recurrent infections (89.3%), microbiological eradication of pathogens (82.3%), and a significant reduction in inflammatory parameters (C-reactive protein, CRP: 88.1%; procalcitonin, PCT: 98.9%). Details of the underlying pathogens can be found in Table A2.

### 2.2. Fosfomycin Therapy and TDM Measurements

The median fosfomycin dose administered in the first 24 h of treatment across all groups was 15 g. A total of 404 TDM measurements were performed, with 143 patients undergoing an initial measurement and 92 (64.3%) of them receiving at least one follow-up measurement. Thirty patients had a fosfomycin plasma concentration of <64 mg/L, 32 of 64–128 mg/L, and 81 of >128 mg/L at initial determination. TDM-guided dosing adjustments resulted in changes in varying fosfomycin concentration ranges (<64 mg/L: from 21% to 14.7%, *p* = 0.208; 64–128 mg/L: from 22.4% to 51.7%, *p* < 0.001; >128 mg/L: from 56.6% to 33.6%, *p* = 0.004).

### 2.3. Effects of Renal Impairment on Fosfomycin Plasma Concentrations

**Figure 1:**
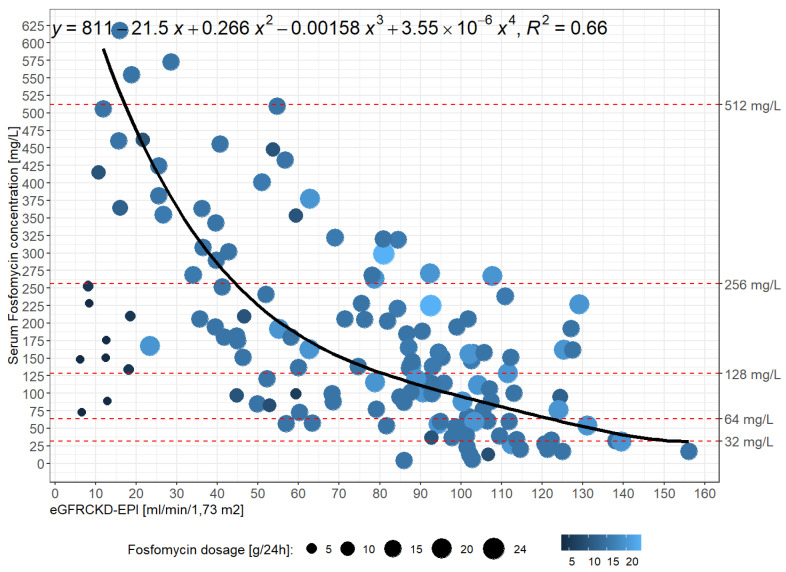
Relationship between initial fosfomycin concentrations and the eGFR, calculated using the CKD-EPI formula. The size and the color gradient of the blue circles indicate the daily fosfomycin dose. R^2^ was calculated using the fitted functions for a daily dose of 15 g.

Initial fosfomycin concentrations exhibited significant inter-individual variability (IQR: 77.2–232.9). Figure 1 demonstrates an exponential correlation between fosfomycin concentration and eGFR values. The distribution of fosfomycin concentrations around the fitted function exhibited high levels of variability (correlation coefficient of R^2^ = 0.66 for a daily dose of 15 g). The mathematical formula for the exponential correlation is y = 811 − 21.5 x + 0.266 x^2^ − 0.00158 x^3^ + 3.55 × 10^−6^ x^4^. Fosfomycin plasma concentrations were >128 mg/L in 45% (36/80) of patients with normal renal function, 70.4% (38/54) of patients with AKI stages I to III, and 77.8% (7/9) of patients with RRT.

**Figure 2:**
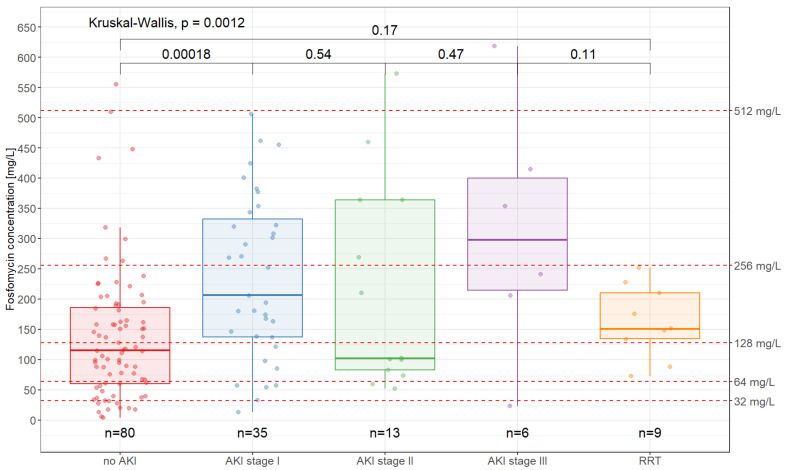
Initial fosfomycin concentrations according to renal function. Renal function was categorized as normal (no AKI), AKI stages I to III, or renal replacement therapy (RRT). Significance was tested using the Wilcoxon test for pairwise comparisons. Multiple comparisons were performed using the Kruskal–Wallis test.

Figure 2 shows the fosfomycin concentration distribution according to renal function. The median fosfomycin concentration was 115 mg/L (IQR: 60.1–185.5) in patients with stable kidney function, 206 mg/L in AKI stage I patients (IQR: 137.2–337.8), 102 mg/L in AKI stage II patients (IQR: 83.0–363.9), and 298 mg/L in AKI stage III patients (IQR: 214.6–399.7).

### 2.4. Adverse Events

Fifty-four percent of patients (77/143) developed AEs. Hypernatremia was the most prevalent AE, followed by hypokalemia and gastrointestinal side effects. The clinical characteristics of patients with AEs are shown in Table 2. There were no significant differences in the initial dose or in the duration of therapy. Patients who experienced AEs had higher fosfomycin plasma concentrations compared to those who did not. The median fosfomycin plasma concentration was higher in patients with AEs vs. no AEs (158 mg/L vs. 131 mg/L, *p* = 0.01). With increasing plasma concentrations (<64 mg/L vs. 64–128 mg/L vs. >128 mg/L), the proportion of patients with AEs increased (37% vs. 56% vs. 59%). No AEs occurred in patients on RRT at an adjusted dose of 3 g. In fosfomycin-based combination therapies, no specific antibiotic was identified as being associated with AEs (Table A3). Significance tests showed no difference for antibiotic groups (*p* = 0.697) and agents (*p* = 0.288).

In addition, a multivariate logistic regression analysis was performed to adjust for all significant parameters from the univariate analyses (*p* < 0.05) that were associated with the development of AEs (Table 3). Age ≥ 70 years (OR 3.70, 95% CI 1.24–11.5; *p* = 0.02) and fosfomycin plasma concentrations > 128 mg/L (OR 3.30, 95% CI 1.09–10.4; *p* = 0.04) were significantly associated with the development of AEs.

## 3. Discussion

In this study, we present real-world data on the use of intravenous fosfomycin-based therapies and the associated AEs. At standard doses, a high variability of fosfomycin plasma concentrations among patients was observed. There was a significant association between high plasma exposure and the occurrence of AEs. Acute renal impairment is common in patient populations with infectious complications and has a strong impact on fosfomycin plasma concentrations. Therefore, the frequent monitoring of renal function appears to be mandatory in patients receiving intravenous fosfomycin treatment.

Since the European Committee on Antimicrobial Susceptibility Testing (EUCAST) no longer specifies clinical breakpoints for fosfomycin susceptibility testing in combination therapies as of 1 January 2024 [23,24], we could no longer use MIC values as target parameters. Instead, the epidemiological cut-off values (ECOFFs) for *Staphylococcus aureus* (32 mg/L [25]) had to be used as multiples to categorize plasma concentration ranges. In clinical practice, TDM determination has proven most reliable when performed from a trough level prior to long-term infusion. A study by Al Jalali et al. [26] supports this approach. They found that intermittent infusion was non-inferior to continuous infusion up to an MIC of 32 mg/L in terms of %T > MIC [26]. Similarly, a Monte Carlo simulation for dose optimization in the treatment of complex osteoarticular infections showed that 8 g of fosfomycin administered as a long-term infusion was equally effective against Gram-negative pathogens with an MIC ≤ 32 mg/L [17]. In this context, TDM-guided dose adjustment in our study was considered as time-dependent killing activity (PK/PD index: T > MIC) [15,26,27,28], and the range of 64–128 mg/L was defined as the target range. A recent study also suggests that an AUC/MIC > 66.6 should be aimed for at an MIC of 32 mg/L, corresponding to a Css of 88.8 mg/L [29].

In addition, we determined factors that influence the plasma concentration of fosfomycin. As a hydrophilic drug, fosfomycin is almost completely eliminated by glomerular filtration. The relationship between the initial fosfomycin concentration and the eGFR showed an exponential correlation between increasing fosfomycin concentration and decreasing renal function (R^2^ = 0.66) and is comparable to the correlation found by König et al. with R^2^ = 0.63 [30]. Furthermore, both this study and our own investigations showed a high variability in plasma concentrations (IQR: 163–438; [30] and 77.2–232.9, respectively). Parker et al. explained the significant pharmacokinetic variability of fosfomycin that they found in critically ill patients with the observed fluctuations in renal function [31]. Acute renal dysfunction frequently occurs in patients with infectious complications and, therefore, has a strong influence on fosfomycin plasma concentrations. In the present study, these were > 128 mg/L in 70.4% (38/54) of the patients with AKI stages I to III and in 77.8% (7/9) of the patients receiving RRT. The median fosfomycin concentration increased from 115 mg/L in patients with stable renal function to 206 mg/L in patients with AKI stage I and 298 mg/L in patients with AKI stage III (*p* < 0.001). This underscores the need for careful monitoring of fosfomycin dosage and regular assessment of renal function in patients with signs of acute renal impairment. In critically ill patients or patients with sepsis, the drug distribution may be altered due to capillary leak syndrome, which affects plasma concentrations. The tendency towards underdosing in patients with normal renal function and overdosing in patients with renal impairment observed in our study is consistent with the results of studies with predominantly renally eliminated beta-lactam antibiotics [32,33,34]. Increased renal clearance has also been identified as a risk factor for low drug concentrations [35,36,37]. In addition, co-administration with other drugs may affect fosfomycin concentrations by altering the distribution or elimination of fosfomycin. Although fosfomycin is mainly excreted unchanged in the urine, individual variations in enzymatic activity may affect plasma concentrations. Differences in drug transporters or minor metabolic pathways may also play a role. In contrast to many other antibiotics, fosfomycin is not subject to hepatic metabolism. When evaluating the antibiotic combinations in our study, the potential risk of drug–drug interactions was also considered. Beta-lactam antibiotics were used most frequently due to their proven synergistic effect (62.2%).

In this study, we found that side effects occurred in 53.8% (77/143) of the patients. The most common AEs were hypernatremia (42.6%), hypokalemia (39.9%), and gastrointestinal symptoms (19.6%). These results are consistent with other studies in which hypernatremia was found in 24% to 47% of patients [30,38,39] and hypokalemia in 22% to 62% of patients [12,38,40,41,42,43]. Hypernatremia is easily explained by the additional sodium content of fosfomycin. Hypokalemia is thought to be due to increased potassium excretion in the distal part of the renal tubules [5,12]. The high sodium content of fosfomycin preparations increases sodium delivery to the aldosterone-sensitive distal nephrons, where increased sodium influx leads to increased potassium excretion [44]. This pathophysiology could suggest therapeutic alternatives to potassium supplementation using mineralocorticoid receptor antagonists (e.g., spironolactone) or the epithelial sodium channel blocker amiloride. In addition, our study demonstrated that as the fosfomycin plasma concentrations increased (<64 mg/L vs. 64–128 mg/L vs. >128 mg/L), the proportion of patients with AEs also increased (37% vs. 56% vs. 59%). This correlation is consistent with the results of a study by Biscarini et al. [39], even if it did not reach statistical significance. With the exception of patients who developed hypernatremia (the most commonly reported AE), the median Cmin value was also significantly higher in patients with AEs (419.5; 266.0–655.0 mg/L) compared to patients without AEs (140.6; 64.9–227.2 mg/L) (*p* = 0.012) [39]. Using multivariate logistic regression, we analyzed the factors associated with the development of AEs during treatment. We found that age ≥ 70 years (OR 3.70, 95% CI 1.24–11.5; *p* = 0.02) and fosfomycin plasma concentrations > 128 mg/L (OR 3.30, 95% CI 1.09–10.4; *p* = 0.04) were significantly associated with the development of AEs.

### Strengths and Limitations

The strength of this study lies in the three-digit number of patients treated with fosfomycin. In addition, the study reflects a clinical setting that describes the determination of fosfomycin plasma concentrations under real-life conditions in a tertiary care hospital. However, due to the substantial variation in fosfomycin plasma concentrations observed in patients receiving standard doses, further research is required.

The retrospective design of the routine clinical data and the monocentric nature of the analysis are major limitations of the study. Study data were unbalanced with respect to the defined concentration ranges, which limited the scope of the comparative analysis and the significance of the statistical analysis. In addition, only trough plasma concentrations of fosfomycin were determined as a reliable determinant in daily clinical practice. The use of combination therapy must also be considered as a potential confounding factor.

## 4. Materials and Methods

### 4.1. Study Design and Patients

We conducted a monocentric retrospective study with routine data. All adult patients treated with intravenous fosfomycin for non-urinary tract infections between 1 January 2021 and 31 December 2023 who received at least one fosfomycin plasma determination were included. Chronic kidney disease (CKD) was defined as a sustained eGFR of <60 mL/min/1.73 m^2^. The eGFR was calculated according to the Chronic Kidney Disease Epidemiology Collaboration (CKD-EPI) formula [45]. AKI was diagnosed and graded according to the Kidney Disease: Improving Global Outcomes (KDIGO) criteria [46]. Gastrointestinal side effects (nausea, vomiting, and/or prescription of antiemetics) and electrolyte imbalances such as hypernatremia (defined as sodium > 145 mmol/L) and hypokalemia (defined as potassium < 3.6 mmol/L) were documented as AEs. Clinical outcomes were hospital discharge, absence of recurrent infection (no hospital readmission within three months due to suspected or proven recurrence of infection), a significant reduction in CRP (reduction in the maximum value by at least 75% [47]), and PCT (reduction in the maximum value by at least 80% [48]), as well as microbiological eradication (defined as the negative microbiological findings of the treated pathogen in a comparable material).

The standard daily dose of fosfomycin was 3 × 5 g intravenously, in accordance with internal clinical guidelines. Dose adjustment for renal function was made according to the recommendations in the Summary of Product Characteristics (SmPC) for patients with a creatinine clearance of less than 40 mL/min [10]. Patients undergoing chronic intermittent dialysis received 3 g of fosfomycin at the end of each dialysis session. Fosfomycin was used only in combination with other antibacterial agents (Table A3). Plasma concentrations were determined as trough levels 30 min before the fourth application after the initial dose and after dose adjustment, respectively. Three groups were formed to investigate the causes and effects of the different fosfomycin plasma concentrations: <64 mg/L, 64–128 mg/L, and >128 mg/L. TDM-guided dose adjustment and subsequent monitoring were managed by infectious disease (ID) specialists. The ID specialists considered a time-dependent killing activity (PK/PD index: T > MIC) based on previous studies [15,26,27,28] and the microbiological characteristics of our cohort, with most bacterial isolates being Staphylococcus aureus and Escherichia coli (Table A2).

### 4.2. TDM Analytics

Fosfomycin concentrations were determined using liquid chromatography with tandem mass spectrometry (LC-MS/MS) [49] provided by the Institute for Biomedical and Pharmaceutical Research (IBMP) in Nuremberg, Germany, using an API 5500 triple quadrupole mass spectrometer (SCIEX, Concord, ON, Canada). Next, 25 µL of each sample was deproteinized with 375 µL internal standard solution (fosfomycin-13C3), subsequently vortex-shaken and centrifuged. The supernatant was further diluted with a mixture of ammonium formate buffer/water/acetonitrile (5:5:90), and 20 µL of each sample was injected into the LC-MS/MS system. Quantification of fosfomycin was performed using a peak area ratio of analyte to internal standard. The linearity of the calibration curve for fosfomycin was proven to be from 0.700 to 410 µg/mL. The lower limit of quantification in plasma was set to 0.700 µg/mL. Samples with concentrations above 410 µg/mL were diluted 1:5 with drug-free human plasma and analyzed again. No interferences were observed for fosfomycin and the internal standards in human plasma. The intra- and inter-day precision of the spiked quality control samples was below 5%, with an intra- and inter-day accuracy between 90% and 110%.

### 4.3. Statistical Analysis

Means and standard deviations (SDs) or medians and interquartile ranges (IQRs) for continuous variables and numbers and percentages for categorical variables were calculated to describe patient characteristics. Significance testing was performed using the chi-square test or Fisher’s exact test for categorical variables and *t*-tests for continuous variables in the case of normally distributed data, or the Wilcoxon test otherwise. Q–Q plots were used to check whether the data were normally distributed. We considered a *p*-value less than or equal to 0.05 to indicate statistical significance. Multiple comparisons were performed with an ANOVA test in the case of normally distributed data or with the Kruskal–Wallis test otherwise. Multivariate logistic regression analysis was performed to evaluate the influence of multiple factors on the development of AEs, considering all parameters that were significant in univariate analyses (*p* < 0.05). R statistical software (version 4.4.2) was used for all statistical analyses.

## 5. Conclusions

At standard doses, a high variability of fosfomycin plasma concentrations was observed among patients. There was a significant correlation between high plasma exposure and the occurrence of AEs. In particular, the effects of acute renal insufficiency on fosfomycin plasma concentrations should be considered. Individualized fosfomycin dosing based on TDM and the intensive monitoring of renal function contribute to reducing drug-related side effects.

## Figures and Tables

**Table 1:** Demographic and clinical characteristics of patients treated with fosfomycin at Hospital St. Georg from 2021 to 2023. Stratification into three groups was based on the fosfomycin plasma concentration range.

Fosfomycin Plasma Concentration	<64 mg/L	64–128 mg/L	>128 mg/L	*p* Value ^a^	*p* Value ^b^	Overall
Number of patients (n)	30 (21.0)	32 (22.4)	81 (56.6)	0.7995	<0.001	143
Age (years)	54 (42–78)	74 (63–81)	74 (64–82)	0.004	0808	73 (60–81)
Male sex (n)	26 (86.7)	22 (68.8)	47 (58.0)	0.167	0.401	95 (66.4)
BMI	26 (23–32)	27 (24–33)	27 (24–31)	0.284	0.528	27 (24–32)
CCI	2 (1–5)	5 (4–7)	6 (4–7)	0.001	0.529	5 (3–7)
eGFR	107 (95–122)	94 (86–108)	74 (42–96)	<0.001	<0.001	93 (67–108)
Length of hospital stay (days)	26.5 (18–44)	39.5 (29–50)	39.5 (25–61)	0.038	0.935	36 (24–56)
In-hospital death (n)	1 (3.3)	1 (3.1)	20 (24.7)	1.000	0.017	22 (15.4)
Community-acquired infection (n)	24 (80.0)	22 (68.8)	52 (64.2)	0.471	0.811	98 (68.5)
Fosfomycin therapy and TDM measurements						
Start dosage (gram)	15 (15–15)	15 (15–16)	15 (15–16)	0.947	0.830	15 (15–16)
Duration of therapy (days)	13 (8–23.5)	17.5 (11–23)	15 (8.7–25)	0.378	0.898	15 (9–25)
Patients with control measurements	15 (50.0)	20 (62.5)	57 (70.4)	0.398	<0.001	92 (64.3)
Count of control measurements	45 (17.2)	125 (47.9)	91 (34.9)	0.003	<0.001	261
Patients after TDM-guided dose adjustment	21 (14.7)	74 (51.7)	48 (33.6)	<0.001	0.0186	143
Assessment of clinical success						
Significant reduction of CRP ^c^	27 (90.0)	30 (93.8)	69 (85.2)	0.940	0.353	126 (88.1)
Significant reduction of PCT ^d^	20 (100.0)	19 (100.0)	50 (98.0)		1.000	89 (98.9)
Absence of recurrent infection ^e^	28 (96.6)	28 (90.3)	52 (85.2)	0.654	0.722	108 (89.3)
Microbiological pathogen eradication ^f^	35 (94.6)	33 (71.7)	86 (86.0)	0.022	0.100	154 (82.3)
Influence of renal function and renal replacement therapy						
Normal renal function (no AKI)	22 (73.3)	22 (68.8)	36 (44.4)	0.317	0.023	80 (55.9)
AKI stage I	5 (16.7)	3 (9.4)	27 (33.3)			35 (24.5)
AKI stage II	2 (6.7)	5 (15.6)	6 (7.4)			13 (9.1)
AKI stage III	1 (3.3)	0 (0.0)	5 (6.2)			6 (4.2)
RRT	0 (0.0)	2 (6.2)	7 (8.6)			9 (6.3)
Adverse events (AEs)						
Gastrointestinal	1 (3.3)	7 (21.9)	20 (24.7)	0.072	0.943	28 (19.6)
Hypernatremia	10 (33.3)	18 (56.2)	33 (40.7)	0.683	0.139	61 (42.6)
Hypokalemia	11 (36.7)	13 (40.6)	33 (40.7)	0.683	0.988	57 (39.9)
Total	11 (36.7)	18 (56.2)	48 (59.3)	0.197	0.936	77 (53.8)

Acute kidney injury (AKI) with stages I, II, and III; BMI, body mass index (kg/m^2^); eGFR, estimated glomerular filtration rate (GFR, mL/min/1.73 m^2^); CCI, Charlson comorbidity index; CRP, C-reactive protein; PCT, procalcitonin; RRT, renal replacement therapy. Data are presented as numbers (percentages) or median values (IQR: Q1–Q3). *p* value ^a^: pairwise comparison (<64 mg/L, 64–128 mg/L), *p* value ^b^: pairwise comparison (64–128 mg/L, >128 mg/L), ^c^ Percentage refers to the number of patients with CRP determination (n = 143), ^d^ Percentage refers to the number of patients with PCT determination (n = 90), ^e^ Percentage refers to the number of patients discharged from hospital (n = 121), and ^f^ Percentage refers to the number of pathogens (n = 187).

**Table 2:** Clinical characteristics comparing patients who experienced adverse events (AEs) related to intravenous fosfomycin and those not (no AEs).

	AEs n = 77	No AEs n = 66	*p* Value	Overall
Fosfomycin dosages and duration of therapy				
Start dosage (gram)	15.0 (15.0, 16.0)	15.0 (15.0, 15.0)	0.032	15.0 (15.0, 16.0)
Duration of therapy (days)	16.0 (10.0, 22.0)	14.0 (9.00, 26.00)	0.832	15.0 (9.0, 25.0)
TDM determinations				
Fosfomycin plasma concentration [mg/L]	158.0 (99.7, 270.8)	131.0 (42.2, 209.0)	0.010	150.8 (77.2, 232.9)
Number of TDM determinations	2.0 (1.0, 3.0)	3.0 (1.0, 4.0)	0.035	2.0 (1.0, 4.0)
Patients with TDM-guided dose adjustment	46 (75.4)	38 (80.9)	0.659	84 (77.8)
Patients with NI-guided dose adjustment	69 (89.6)	58 (87.9)	0.951	127 (88.8)
Fosfomycin concentration < 64 mg/mL	11 (14.3)	19 (28.8)	0.101	30 (21.0)
Fosfomycin concentration 64–128 mg/mL	18 (23.4)	14 (21.2)		32 (22.4)
Fosfomycin concentration > 128 mg/mL	48 (62.3)	33 (50.0)		81 (56.6)
Influence of renal function and renal replacement therapy				
Normal renal function (no AKI)	43 (55.8)	37 (56.1)	0.003	80 (55.9)
AKI stage I	25 (32.5)	10 (15.2)		35 (24.5)
AKI stage II	7 (9.1)	6 (9.1)		13 (9.1)
AKI stage III	2 (2.6)	4 (6.1)		6 (4.2)
RRT	0 (0.0)	9 (13.6)		9 (6.3)
Laboratory results				
Sodium [mmol/L]	141.4 (139.0, 144.7)	140.2 (137.4, 143.1)	<0.001	140.9 (138.2, 143.8)
Potassium [mmol/L]	3.5 (3.2, 4.1)	4.2 (3.9, 4.5)	<0.001	3.9 (3.5, 4.4)
NT-proBNP [ng/L]	4283 (2433, 10560)	32,530 (2691, 3715)	0.355	3851 (2442, 9210)
Troponin [µg/L]	50.3 (33.7, 54.6)	39.0 (23.5, 59.0)	0.712	48.7 (27.7, 59.0)
Uric acid [µmol/L]	267 (175, 359)	249 (171, 337)	0.568	267 (174, 357)
Albumin [g/L]	25.1 (21.5, 29.3)	28.5 (24.4, 31.7)	<0.001	26.8 (22.6, 30.2)

AEs, adverse events; acute kidney injury (AKI) with stages I, II, and III; RRT, renal replacement therapy. Data are presented as numbers (percentages) or median values (IQR: Q1–Q3).

**Table 3:** Results of the multivariate logistic regression analysis of risk factors for the development of AEs.

	Univariate Logistic Regression			Multivariate Logistic Regression		
Characteristic	OR	95% CI	*p*-Value	OR	95% CI	*p*-Value
Age group ≥ 70 years	3.62	1.82, 7.40	<0.001	3.70	1.24, 11.5	0.020
Female sex	1.46	0.65, 3.38	0.4			
AKI stage I	1.42	0.61, 3.50	0.4			
Fosfomycin concentration						
64–128 mg/L	2.59	0.93, 7.55	0.073	1.43	0.43, 4.68	0.6
>128 mg/L	4.13	1.67, 10.7	0.003	3.30	1.09, 10.4	0.036
CCI ≥ 4	4.74	2.10, 11.1	<0.001	2.07	0.69, 6.20	0.2
Diabetes mellitus	2.22	1.02, 4.98	0.047	1.76	0.61, 5.26	0.3
Chronic kidney disease CKD 3–5	1.75	0.83, 3.77	0.15			
ICU	0.5	0.19, 1.26	0.14			
Adipositas	1.14	0.45, 2.88	0.8			

OR, odds ratio; CI, confidence interval; AKI stage I, acute kidney injury stage I; CCI, Charlson comorbidity index; ICU, intensive care unit

## Data Availability

Anonymized data will be available upon request. Contact kathrin.marx@sanktgeorg.de.

## Abbreviations

The following abbreviations are used in this manuscript:

AE(s)	Adverse event(s)
AKI	Acute kidney injury
AUC	Area under the curve
BMI	Body mass index
CCI	Charlson comorbidity index
CI	Confidence interval
CKD	Chronic kidney disease
CKD-EPI	Chronic Kidney Disease Epidemiology Collaboration
CRP	C-reactive protein
ECOFF	Epidemiologic cut-off value
eGFR	Estimated glomerular filtration rate
EUCAST	European Committee for Antimicrobial Susceptibility Testing
ICU	Intensive care unit
ID	Infectious diseases
IQR	Interquartile range
KDIGO	Kidney Disease: Improving Global Outcomes
LC-MS/MS	Liquid chromatography with tandem mass spectrometry
MIC	Minimum inhibitory concentration
OR	Odds ratio
PCT	Procalcitonin
PK/PD	Pharmacokinetic/pharmacodynamic
PTA	Probability of target attainment
RR	Relative risk
RRT	Renal replacement therapy
SD	Standard deviation
SmPC	Summary of Product Characteristics
TDM	Therapeutic drug monitoring

## Appendix A

**Table A1:** Baseline characteristics regarding comorbidities and selected laboratory parameters of patients treated with fosfomycin at Hospital St. Georg 2021–2023.

Fosfomycin Plasma Concentration	<64 mg/L	64–128 mg/L	>128 mg/L	*p* Value ^a^	*p* Value ^b^	Overall
Number of patients (n)	30 (21.0)	32 (22.4)	81 (56.6)	0.7995	<0.001	143
Type of infection (n)						
Bone and joint infection	11 (36.7)	16 (51.6)	37 (45.7)	0.263	0.535	64 (45.1)
Skin and soft tissue infection	7 (23.3)	2 (6.5)	15 (18.5)			24 (16.9)
Endocarditis	3 (10)	5 (16.1)	7 (8.6)			15 (10.6)
Sepsis	2 (6.7)	4 (12.9)	10 (12.3)			16 (11.3)
Pneumonia	3 (10.0)	0 (0.0)	3 (3.7)			6 (4.2)
Intracranial abscess	1 (3.3)	1 (3.2)	3 (3.7)			5 (3.5)
Port infection	1 (3.3)	2 (6.5)	1 (1.2)			4 (2.8)
Dialysis catheter	0 (0.0)	1 (3.2)	1 (1.2)			2 (1.4)
Intraspinal abscess	0 (0.0)	0 (0.0)	2 (2.5)			2 (1.4)
Encephalitis	0 (0.0)	0 (0.0)	1 (1.2)			1 (0.7)
Pacemaker	0 (0.0)	0 (0.0)	1 (1.2)			1 (0.7)
Heart valve prosthesis	1 (3.3)	0 (0.0)	0 (0.0)			1 (0.7)
Intraabdominal infection	1 (3.3)	0 (0.0)	0 (0.0)			1 (0.7)
Comorbidities (n)						
Renal disease ^c^	0 (0)	4 (12.5)	19 (23.5)	0.138	0.296	23 (16.1)
Liver disease	2 (6.7)	2 (6.2)	13 (16.0)	1.000	0.282	17 (11.9)
Diabetes mellitus	4 (13.3)	16 (50.0)	29 (35.8)	0.005	0.240	49 (34.3)
Obesity	8 (26.7)	6 (18.8)	13 (16.0)	0.659	0.947	27 (18.9)
Cardiovascular disease	1 (3.3)	10 (31.2)	26 (32.1)	0.011	1.000	37 (25.9)
Hypertension	15 (50.0)	19 (59.4)	39 (48.1)	0.627	0.386	73 (51.0)
Laboratory results						
Creatinin [µmol/L]	56 (47–74)	64 (50–77)	84.5 (63–142)	0.090	<0.001	69 (52, 90)
Sodium [mmol/L]	141 (138–143.2)	140.5 (138.3–143.3)	141.2 (138.7–144.6)	0.686	0.053	141 (138.6–143.8)
Potassium [mmol/L]	3.9 (3.4–4.2)	4.0 (3.6–4.3)	3.9 (3.4–4.4)	0.462	0.927	3.9 (3.5–4.4)
NT-proBNP [ng/L]	2315 (2112–7471)	3539 (2648–6418)	8298 (3466–16,929)	0.614	0.101	4547 (2614–11,706)
Troponin [µg/L]	33.9 (20.4–34.2)	48.7 (31.5–51.3)	51.3 (29.9–59.5)	0.166	0.744	48.1 (25.9–56.5)
Uric acid [µmol/L]	175 (130–211)	214 (173–297)	322 (187–392)	0.095	0.160	226 (166–346)
Albumin [g/L]	26.6 (23.8–31.8)	28.0 (23.2–30.2)	25.5 (21.1–29.8)	0.947	0.054	26.8 (22.6–30.2)

Data are presented as numbers (percentages) or median values (IQR: Q1–Q3). *p* value ^a^: pairwise comparison (<64 mg/L, 64–128 mg/L), *p* value ^b^: pairwise comparison (64–128 mg/L, >128 mg/L), Renal disease ^c^: CKD stages 3–5.

**Table A2:** Microbial isolates identified among the 143 study patients.

MIC for Fosfomycin	≤8 µg/mL	≤16 µg/mL	32 µg/mL	≥64 µg/mL	>256 µg/mL	Overall
N (%)	89 (51.4)	73 (42.2)	6 (3.5)	4 (2.3)	1 (0.6)	173
Gram-negative pathogens						
Enterobacterales	1 (1.1)	67 (91.8)	6 (100.0)	1 (25.0)	0 (0.0)	75 (43.4)
*Escherichia coli*	0 (0.0)	38 (52.1)	0 (0.0)	0 (0.0)	0 (0.0)	38 (22.0)
*Enterobacter cloacae complex*	0 (0.0)	7 (9.6)	3 (50.0)	0 (0.0)	0 (0.0)	10 (5.8)
*Klebsiella pneumoniae*	1 (1.1)	8 (11.0)	0 (0.0)	0 (0.0)	0 (0.0)	9 (5.2)
*Proteus mirabilis*	0 (0.0)	5 (6.8)	0 (0.0)	0 (0.0)	0 (0.0)	5 (2.9)
*Klebsiella aerogenes*	0 (0.0)	3 (4.1)	1 (16.7)	0 (0.0)	0 (0.0)	4 (2.3)
*Klebsiella oxytoca*	0 (0.0)	2 (2.7)	0 (0.0)	1 (25.0)	0 (0.0)	3 (1.7)
*Salmonella* Enteritidis	0 (0.0)	2 (2.7)	0 (0.0)	0 (0.0)	0 (0.0)	2 (1.2)
*Serratia marcescens*	0 (0.0)	0 (0.0)	2 (33.3)	0 (0.0)	0 (0.0)	2 (1.2)
*Escherichia hermannii*	0 (0.0)	1 (1.4)	0 (0.0)	0 (0.0)	0 (0.0)	1 (0.6)
*Citrobacter koseri*	0 (0.0)	1 (1.4)	0 (0.0)	0 (0.0)	0 (0.0)	1 (0.6)
Other Gram-negative pathogens	1 (1.1)	0 (0.0)	0 (0.0)	0 (0.0)	0 (0.0)	1 (0.6)
*Cardiobacterium hominis (HACEK)*	1 (1.1)	0 (0.0)	0 (0.0)	0 (0.0)	0 (0.0)	1 (0.6)
Gram-positive pathogens						
*Staphylococcus aureus*	61 (68.5)	6 (8.2)	0 (0.0)	0 (0.0)	0 (0.0)	67 (38.7)
Coagulase-negative staphylococci	23 (25.8)	0 (0.0)	0 (0.0)	2 (50.0)	0 (0.0)	25 14.4)
*Staphylococcus epidermidis*	20 (22.5)	0 (0.0)	0 (0.0)	0 (0.0)	0 (0.0)	20 (11.6)
*Staphylococcus hominis*	0 (0.0)	0 (0.0)	0 (0.0)	2 (50.0)	0 (0.0)	2 (1.2)
*Staphylococcus lugdunensis*	1 (1.1)	0 (0.0)	0 (0.0)	0 (0.0)	0 (0.0)	1 (0.6)
*Staphylococcus simulans*	1 (1.1)	0 (0.0)	0 (0.0)	0 (0.0)	0 (0.0)	1 (0.6)
*Staphylococcus cohnii* spp. *urealyticus*	1 (1.1)	0 (0.0)	0 (0.0)	0 (0.0)	0 (0.0)	1 (0.6)
Other Gram-positive pathogens	3 (3.4)	0 (0.0)	0 (0.0)	1 (25.0)	1 (100.0)	5 (2.9)
*Enterococcus faecalis*	1 (1.1)	0 (0.0)	0 (0.0)	1 (25.0)	0 (0.0)	2 (1.2)
*Streptococcus intermedius*	1 (1.1)	0 (0.0)	0 (0.0)	0 (0.0)	0 (0.0)	1 (0.6)
*Streptococcus pneumoniae*	1 (1.1)	0 (0.0)	0 (0.0)	0 (0.0)	0 (0.0)	1 (0.6)
*Listeria monocytogenes*	0 (0.0)	0 (0.0)	0 (0.0)	0 (0.0)	1 (100.0)	1 (0.6)

The MIC for fosfomycin was subsequently determined using the bioMérieux VITEK^®®^ 2 microdilution method, in accordance with the current version of the EUCAST recommendations (V13.1 dated 29 June 2023). Data are presented as numbers (percentages).

**Table A3:** Antibiotic groups and differentiation according to active substances as combination partners of fosfomycin therapy and the development of AEs.

Antibiotics	AEs (n = 113)	No-AEs (n = 59)	Overall (n = 172)
Aminoglycosides	1 (0.9)	1 (1.7)	2 (1.2)
Gentamicin	1 (0.9)	0 (0.0)	1 (0.6)
Tobramycin	0 (0.0)	1 (1.7)	1 (0.6)
Beta-lactams	70 (61.9)	37 (62.7)	107 (62.2)
Cefazolin	16 (14.2)	11 (18.6)	27 (15.7)
Cefotaxim	10 (8.8)	3 (5.1)	13 (7.6)
Ampicillin	10 (8.8)	1 (1.7)	11 (6.4)
Ampicillin/Sulbactam	5 (4.4)	5 (8.5)	10 (5.8)
Benzylpenicillin	7 (6.2)	3 (5.1)	10 (5.8)
Flucloxacillin	8 (7.1)	2 (3.4)	10 (5.8)
Meropenem	6 (5.3)	4 (6.8)	10 (5.8)
Piperacillin/Tazobactam	1 (0.9)	5 (8.5)	6 (3.5)
Ceftriaxon	1 (0.9)	0 (0.0)	3 (1.7)
Cefuroxim	1 (0.9)	0 (0.0)	2 (1.2)
Imipenem	2 (1.8)	0 (0.0)	2 (1.2)
Amoxicillin	1 (0.9)	0 (0.0)	1 (0.6)
Ceftazidim	1 (0.9)	0 (0.0)	1 (0.6)
Ceftazidim/Avibactam	1 (0.9)	0 (0.0)	1 (0.6)
Clindamycin	2 (1.8)	1 (1.7)	3 (1.7)
Cotrimoxazole	8 (7.1)	6 (10.2)	14 (8.1)
Daptomycin	4 (3.5)	0 (0.0)	4 (2.3)
Fluoroquinolones	5 (4.4)	3 (5.1)	8 (4.7)
Ciprofloxacin	2 (1.8)	2 (3.4)	4 (2.3)
Levofloxacin	3 (2.7)	1 (1.7)	4 (2.3)
Rifampicin	6 (5.3)	1 (1.7)	7 (4.1)
Tetracyclines	12 (10.6)	9 (15.3)	21 (12.2)
Doxycyclin	8 (7.1)	7 (11.9)	15 (8.7)
Tigecyclin	3 (2.7)	1 (1.7)	4 (2.3)
Minocyclin	1 (0.9)	1 (1.7)	2 (1.2)
Vancomycin	5 (4.4)	1 (1.7)	6 (3.5)

Data are presented as numbers (percentages). The significance test showed no difference for antibiotic groups (*p* = 0.697) and agents (*p* = 0.288).
